# Supplementary material for: A large Australian longitudinal cohort registry demonstrates sustained safety and efficacy of oral medicinal cannabis for at least two years
Source: PLoS One. 2022 Nov 18;17(11):e0272241. doi: 10.1371/journal.pone.0272241 (PMC9674134; doi:10.1371/journal.pone.0272241)
Supplement: S1 Data — (ZIP) [file pone.0272241.s001.zip › Data/ReadMe.pdf]

This document contains the instructions on how to open and use the datasets within the Data folder to generate the tables and figures displayed in the manuscript. The datasets used include individual level patient- and clinician-reported variables from the Australian Emyria Clinical e-Registry (AECeR). Specifically, the Data folder contains the following items:

- Figure2_dataset.dta
- Figure3_dataset.dta
- Figure4_dataset.dta
- ReadMe.pdf
- Replication_code.R
- Table2_dataset.dta
- Table3_dataset.dta
- Table4_dataset.dta
- Table5_and_Figure1_dataset.dta
- Table6_dataset.dta
- Table7_dataset.dta

The replication process proceeds as follows. To begin, open Replication_code.R and run code lines 5–10 to load the required packages. Next, execute lines 15–25 to open the various datasets (as listed above) into the current session, before running code lines 30–93 in order to create the necessary variables for the analysis. Finally, execute lines 99–590 to generate the results displayed in both the tables as well as figures of the manuscript.[1] Notice that code for Table 1 is not included in Replication_code.R because it is constructed manually using information made available by the Therapeutic Goods Administration (TGA).

[1]Note that copies of all figures produced are also stored in the corresponding Figure folder contained in the Supporting Information file provided.
